# Supplementary material for: Self-supervised learning with application for infant cerebellum segmentation and analysis
Source: Nat Commun. 2023 Aug 5;14:4717. doi: 10.1038/s41467-023-40446-z (PMC10404262; doi:10.1038/s41467-023-40446-z)
Supplement: Supplementary file 1 — Supplementary Information [file 41467_2023_40446_MOESM1_ESM.pdf]

Self-Supervised Learning with Application for Infant Cerebellum Segmentation and Analysis

Yue Sun[1], Limei Wang[1], Kun Gao[1], Shihui Ying[1], Weili Lin[1], Kathryn L. Humphreys[2,3], Gang Li[1], Si-jie Niu[1], Mingxia Liu[1*], Li Wang[1*]

Supplementary Note 1. Manual segmentation protocol for infant cerebellum MRIs

Manual (reference) segmentations of the cerebellum were generated based on T1- and T2-weighted images using ITK-SNAP (www.itksnap.org) (version 3.8.0) through mouse painting of cerebellar voxels in all slices. Each voxel was assigned to only one of the three tissue categories: cerebrospinal fluid (CSF), gray matter (GM) and white matter (WM), as illustrated in Supplementary Fig. 1. To ensure consistency and smoothness, observers adjusted the tissue contrast to maximally differentiate tissues, and checked the results in 3 orthogonal views and a surface rendering view. Our approach involved identifying WM first, followed by CSF, with the remaining voxels being classified as GM. The manual segmentations were performed step by step to ensure accuracy as follows:

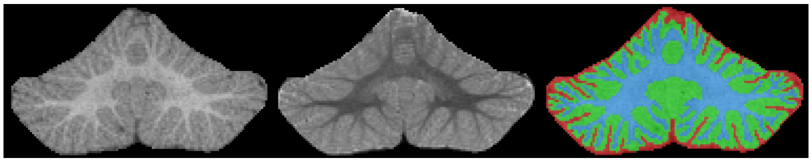

Supplementary Fig. 1. T1- and T2-weighted images and the corresponding manual labels (red: CSF; green: GM; blue: WM).

In *Step 1*, we focus on identifying the arbor vitae (cerebellar WM), which has a branched and folded tree-like appearance without any "hole" or "handle", as depicted in Supplementary Fig. 2. To enhance the tissue contrast, we use the "Curve-Based Contrast Adjustment" tool embedded in ITK-SNAP (as shown in Supplementary Fig. 3). To access this tool, navigate to *Tools*→ *Image Contrast*→ *Contrast Adjustment*. Furthermore, we utilize the "3D rendering" tool in ITK-SNAP to detect any errors in the segmentation. As illustrated in Supplementary Fig. 4, an error is noticeable in the first row of the "3D rendering" view (indicated by a white cross-hair), which is then corrected in the second row. We also provide an example of the manual result for the WM in Supplementary Fig. 5.

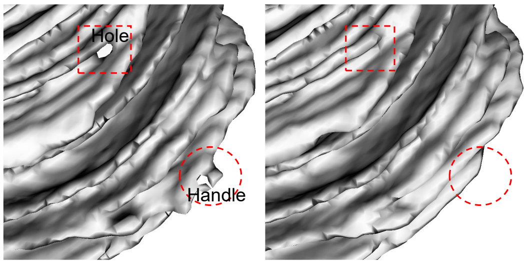

Supplementary Fig. 2. Topological errors of cerebellum segmentations such as the shapes of "hole" or "handle" (left) and the corresponding manual label (right).

In *Step 2*, we identify the CSF, which is the outermost tissue layer and always lies at least one voxel distance from the WM. During the annotation process, it is crucial to adjust the tissue contrast of both T1w and T2w images to clearly identify the CSF. To do this, we make use of the "Curve-Based Contrast Adjustment" tool embedded in ITK-SNAP, as depicted in Supplementary Fig. 3. Supplementary Fig. 6 provides an example of the manual result for the CSF.

In *Step 3*, the remaining voxels are GM, with results shown in Supplementary Fig. 7.

[1] Department of Radiology and Biomedical Research Imaging Center, University of North Carolina at Chapel Hill, NC 27599, USA.
[2] Department of Psychology and Human Development, Vanderbilt University, TN 37203, USA.
[3] Department of Psychiatric and Behavioral Sciences, School of Medicine, Tulane University, LA 70118, USA.
[*] Corresponding authors: M. Liu (mxliu@med.unc.edu) and L. Wang (li_wang@med.unc.edu).

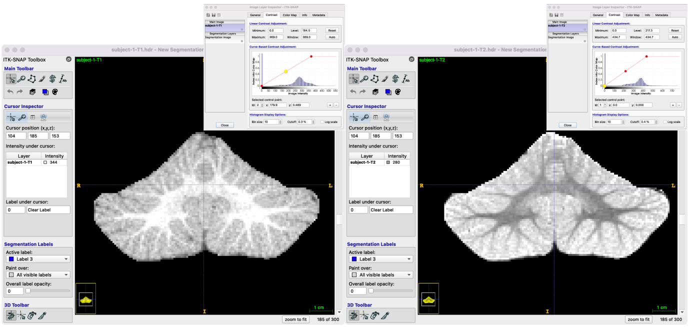

Supplementary Fig. 3. Adjusting the tissue contrast using the embedded "Curve-Based Contrast Adjustment" tool (can be found in Tools→Image Contrast→Contrast Adjustment) in ITK-SNAP.

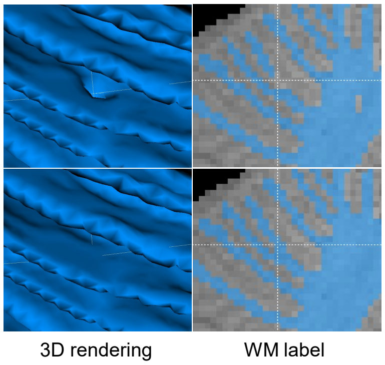

Supplementary Fig. 4. Label correction assisted by "3D rendering" tool in ITK-SNAP.

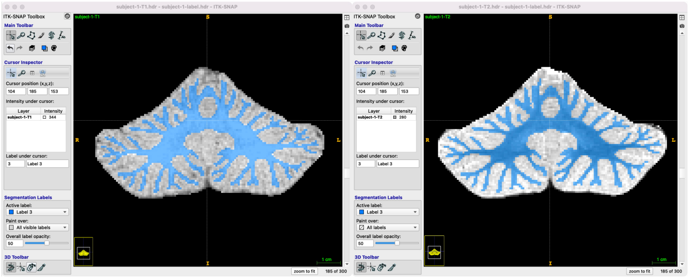

Supplementary Fig. 5. Cerebellar WM manual segmentation result (in blue).

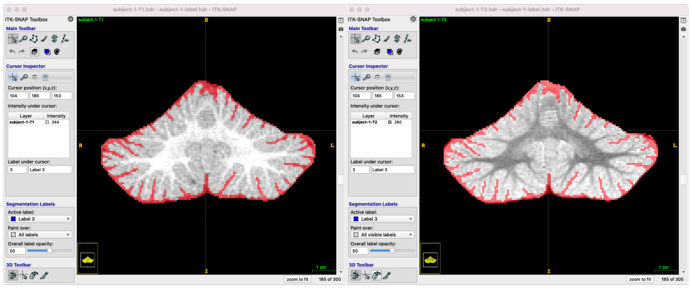

Supplementary Fig. 6. Cerebellar CSF manual segmentation result (in red).

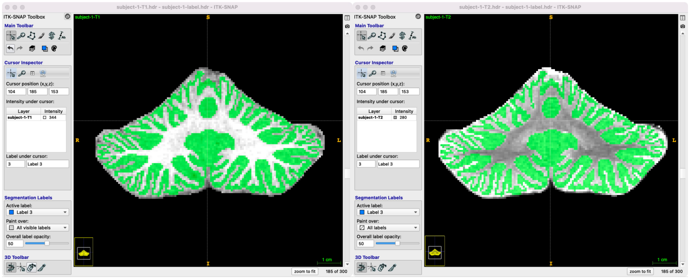

Supplementary Fig. 7. Cerebellar GM manual segmentation result (in green).

Supplementary Note 2. Validation in source domain (24-month-old subjects)

We have made quantitative validation on target domains from different time-points and sites in the main manuscript. In the following, we tested the proposed method in the source domain via a 2-fold cross-validation with 10 repetitions. Specifically, in each repetition, eighteen 24-month-old subjects were randomly partitioned into two folds, with 9 subjects in each fold. Each fold was alternatively used as training subjects, while the other fold was treated as testing subjects. For comparison, we only chose the ADU-Net since it had previously demonstrated the best performance among the competing methods in Table 1 of the main manuscript. In addition to the 9 subjects used for training, the proposed SSL method also utilized automatically-generated reliable samples from unlabeled testing subjects for training. The results showed that the Dice ratios (%, Mean$\pm$Standard Deviation) in 10 repetitions achieved by the ADU-Net were 91.50±1.26, 90.92±1.19, 92.55±0.94 for CSF, GM and WM, respectively. The proposed SSL method achieved slightly improved performance, with Dice ratios of 92.44±1.25, 91.39±1.10, and 93.09±1.73 for CSF, GM, and WM, respectively. This experiment demonstrates that the automatically-generated reliable samples can also boost the segmentation performance in the source domain.

Supplementary Note 3. Influence of gradual label propagation

Segmenting the cerebellum of younger infants, such as 0-month-old subjects, poses a significant challenge due to the substantial distribution gap between these subjects and 24-month-old infants. To address this issue, we propose a gradual label propagation strategy in SSL that gradually propagates labels from 24- to 0-month-old subjects (i.e., $24 \rightarrow 18 \rightarrow 12 \rightarrow 9 \rightarrow 6 \rightarrow 3 \rightarrow 0$). In order to evaluate the effectiveness of this approach, we compared it with the direct propagation strategy (i.e., $24 \rightarrow 0$) in terms of segmentation accuracy.

We present the results of this comparison in Supplementary Fig. 8, which shows the segmentation results of a representative 0-month-old subject obtained with our SSL model using direct and gradual propagation strategies. Our findings indicate that the direct propagation strategy does not yield accurate segmentations due to the significant distribution difference between 24- and 0-month-old subjects. In contrast, the proposed gradual propagation strategy outperforms the direct propagation strategy, leading to much more accurate segmentation outcomes. These results demonstrate the effectiveness of our proposed approach in addressing the distribution gap between different time points.

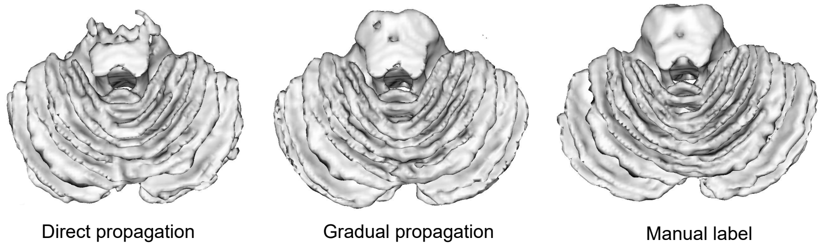

Supplementary Fig. 8. Visual comparison of segmentation results yielded by our SSL using different propagation strategies for one representative 0-month-old subject. The segmentation results are obtained by SSL with direct propagation (left), SSL with gradual propagation (middle), and the manual label (right).

Supplementary Note 4. Influence of the spatially-weighted cross-entropy loss

To demonstrate the advantage of the proposed spatially-weighted cross-entropy loss, we compared it with the conventional cross-entropy loss. In Supplementary Fig. 9, we reported the WM segmentations achieved by our method with only the conventional cross-entropy loss and the proposed spatially-weighted cross-entropy loss in

zoomed views, as well as corresponding Dice ratio and HD95 evaluations. We can see from Supplementary Fig. 9a with the conventional cross-entropy loss, there are many topological errors (as indicated by red arrows in the first column), whereas these errors are largely avoided by leveraging our designed loss (as shown in the second column). The same conclusion can also be summarized from Supplementary Fig. 9b, according to higher Dice ratios and lower HD95 values produced by our proposed loss.

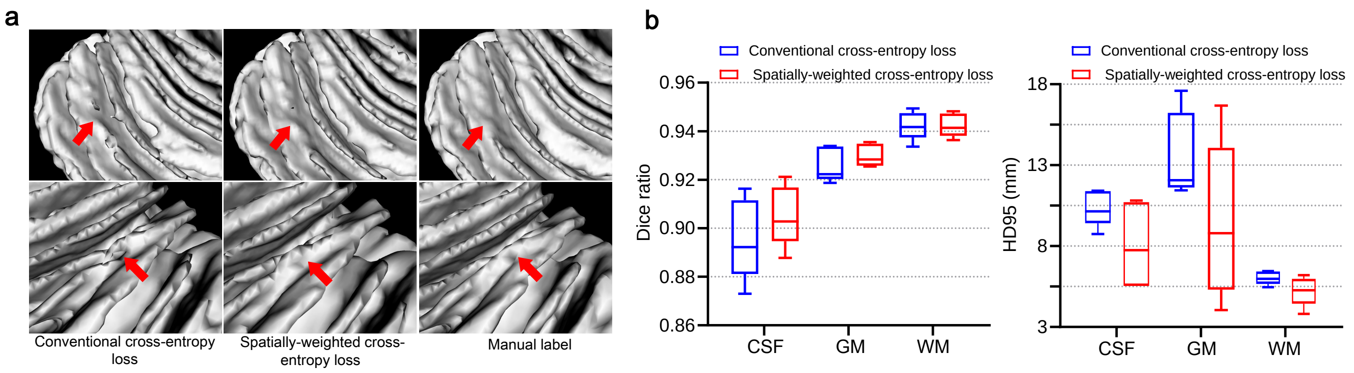

Supplementary Fig. 9. Accuracy improvements by the proposed spatially-weighted cross-entropy loss. a, Results achieved by our SSL with the conventional cross-entropy loss (left) and the proposed spatially-weighted cross-entropy loss (middle) in WM segmentations, and the corresponding manual labels (right). **b**, Quantitative comparisons between the proposed spatially-weighted cross-entropy loss and conventional cross-entropy loss, in terms of Dice ratio and HD95 on 5 testing subjects from BCP at 12 months. In each box plot, the midline represents the median value, and its lower and upper edges represent the first and third quartiles. The whiskers go down to the smallest value and up to the largest. Source data are provided as a Source Data file.

Supplementary Note 5. Validation of the confidence model

The performance of the confidence model is crucial in generating reliable training samples in the target domain. To analyze the performance of the confidence model, we conducted a 2-fold cross-validation experiment (with 10 repetitions) to quantitatively measure the Dice ratio between the predicted and true confidence maps for eighteen 24-month-old subjects. In each repetition, the eighteen subjects were randomly split into two folds, with each fold being alternatively used for training and testing. Supplementary Fig. 10 displays the Dice ratios of the predicted confidence maps for each repetition, and it is evident that most Dice ratio values are above 90%, demonstrating that the predicted confidence maps can effectively detect the reliability of automated segmentations.

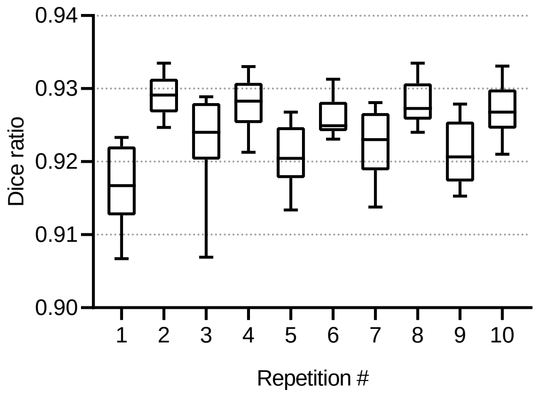

Supplementary Fig. 10. Evaluation on predicted confidence maps on 18 subjects at 24 months by performing a 2-fold cross-validation with 10 repetitions. In each box plot, the midline represents the median value, and its lower and upper edges represent the first and third quartiles. The whiskers go down to the smallest value and up to the largest. Source data are provided as a Source Data file.

To further illustrate the impact of confidence maps, we performed an ablation experiment to compare the difference in target-domain-specific segmentation models trained with and without confidence maps. With confidence maps, we automatically generated a set of reliable training samples and trained a target-domain-specific segmentation model for each domain using the proposed spatially-weighted cross-entropy loss. Without confidence maps, we trained a target-domain-specific segmentation model for each domain using all testing data as training samples and conventional cross-entropy loss. There were 45 testing subjects with manual labels at five target domains (40 BCP subjects at four time-points and 5 Philips subjects at six months of age). We trained five target-domain-specific segmentation models and reported the corresponding Dice ratios in Supplementary Fig. 11. The results with

confidence maps (red) were more accurate and significantly better than the results without confidence maps (blue) for most age groups, as shown in Supplementary Fig. 11 and Supplementary Table 1.

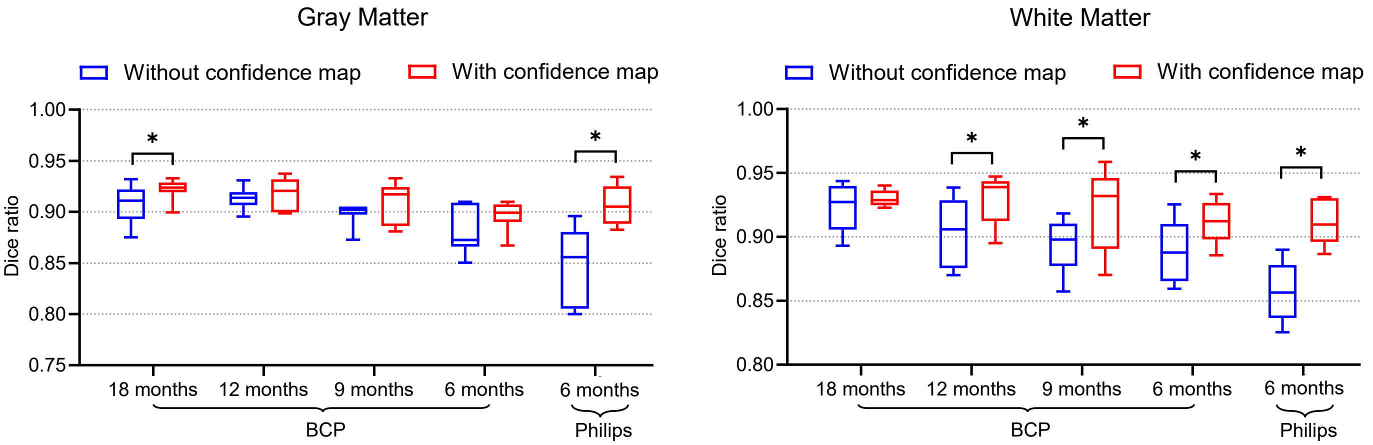

Supplementary Fig. 11. Evaluation on automated segmentations for 45 cross-time-point testing subjects obtained by corresponding target-domain-specific segmentation models trained without/with confidence maps, with $*$ indicating p-value < 0.05 (Wilcoxon signed rank test, two-sided). The exact p-values are available in Supplementary Table 1. In each box plot, the midline represents the median value, and its lower and upper edges represent the first and third quartiles. The whiskers go down to the smallest value and up to the largest. Source data are provided as a Source Data file.

Supplementary Table 1. P**-values (Wilcoxon signed rank test, two sided) between segmentation results of models trained with/without confidence maps, with $*$ indicating p-value < 0.05.**

	BCP				Philips
Age (in month)	18	12	9	6	6
GM	0.0371*	0.2324	0.1934	0.1055	0.0119*
WM	0.2324	0.0330*	0.0488*	0.0417*	0.0020*

Supplementary Note 6. Application on cross-site infant cerebrum segmentation

Our framework is highly versatile and can be extended to other tasks, particularly those that involve multi-site datasets. To further validate the efficacy of our proposed SSL method, we applied it to the infant cerebrum segmentation task in our organized iSeg-2019 challenge. In this challenge, the training subjects were from the Multi-visit Advanced Pediatric Brain Imaging Study[1], while the testing subjects were from three different sites with distinct imaging protocols and scanners, namely UNC/UMN (BCP) with a Siemens scanner, Stanford University with a GE scanner, and Emory University with a Siemens scanner, as described in[2]. The data collection sites obtained study protocol approval from their respective Institutional Review Boards, and all participating subjects had informed consent provided by their parent or legal guardian. To compare our method with the top three methods in the challenge, namely QL111111, Tao_SMU, and FightAutism, we conducted an experiment and the segmentation results are shown in Supplementary Fig. 12. The results indicate that our proposed method generated more consistent and accurate segmentations, as compared to the competing methods. This finding is also supported by the quantitative results in the top panel of Supplementary Fig. 12, which show that our method achieved the highest Dice ratio. Moreover, our method demonstrated the most consistent performance across the three different sites, thereby validating its effectiveness in addressing the domain shift problem.

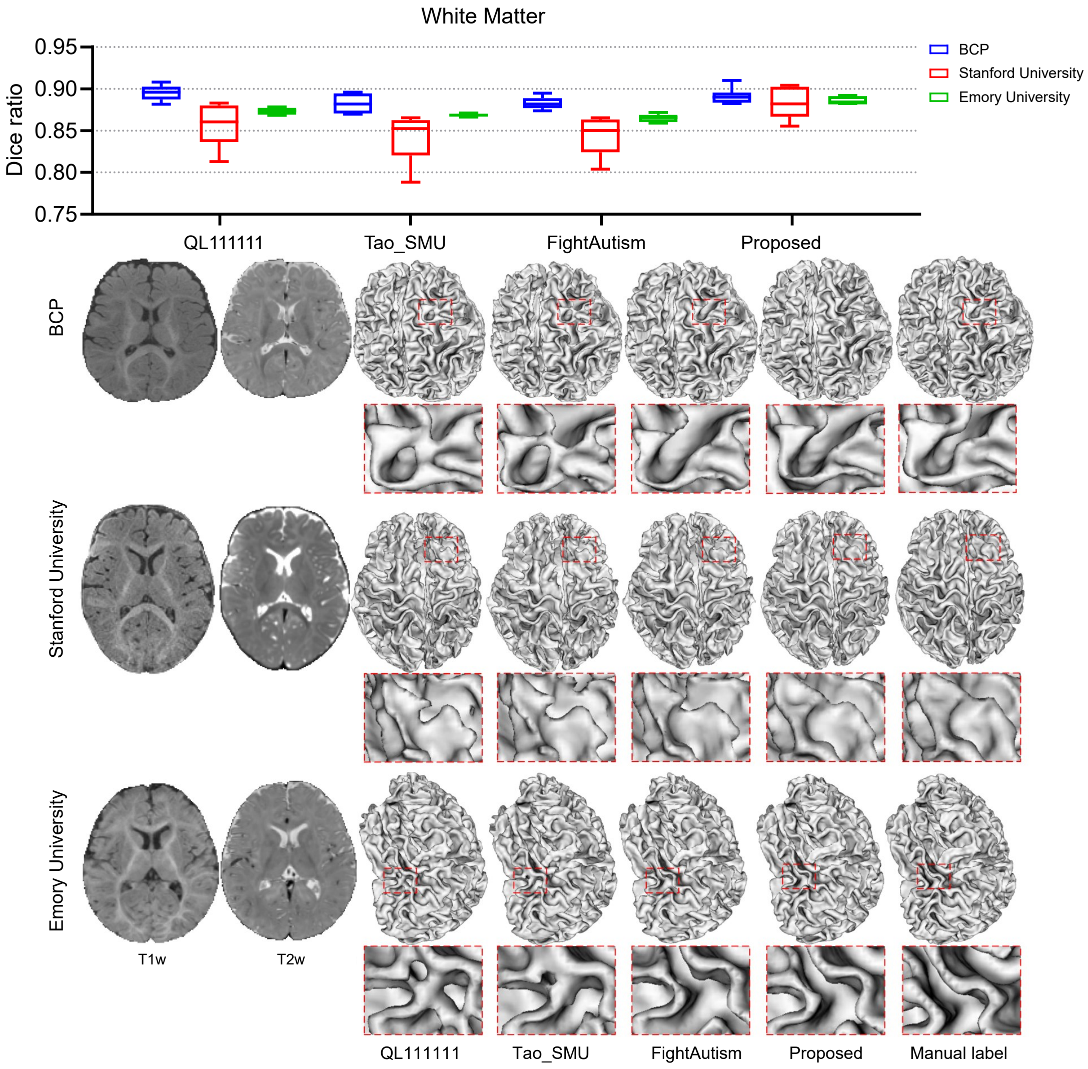

Supplementary Fig. 12. Segmentation results of top 3 methods and the proposed method on iSeg-2019 testing subjects from three sites, i.e., UNC/UMN (BCP), Stanford University, and Emory University, with the corresponding Dice ratio values (BCP: 6 subjects, Stanford University: 5 subjects, Emory University: 5 subjects). Zoomed views are provided for a better visualization. In each box plot, the midline represents the median value, and its lower and upper edges represent the first and third quartiles. The whiskers go down to the smallest value and up to the largest. Source data are provided as a Source Data file.

Supplementary Note 7. Influence of the number of training samples

In the proposed self-supervised training strategy, confidence maps are used to generate a set of reliable training samples for each target domain. In this study, we investigated the impact of the number of training samples on the segmentation performance of 18-month-old subjects, through a cross-validation. We randomly selected different numbers of samples from each of the 10 unlabeled testing subjects and used them to train a segmentation model. This model was then applied to another set of 10 testing subjects (with manual labels) for a quantitative evaluation. Specifically, we extracted 250, 500, 750, 1,000, 1,250 and 1,500 training samples from each of the 10 unlabeled testing subjects and evaluated the corresponding Dice ratio results for GM and WM. As shown in Supplementary Fig. 13, the Dice ratios gradually increase as the number of training samples increases, which is expected. Based on the tradeoff between accuracy and training complexity, we recommend extracting 1,000 training samples from each subject.

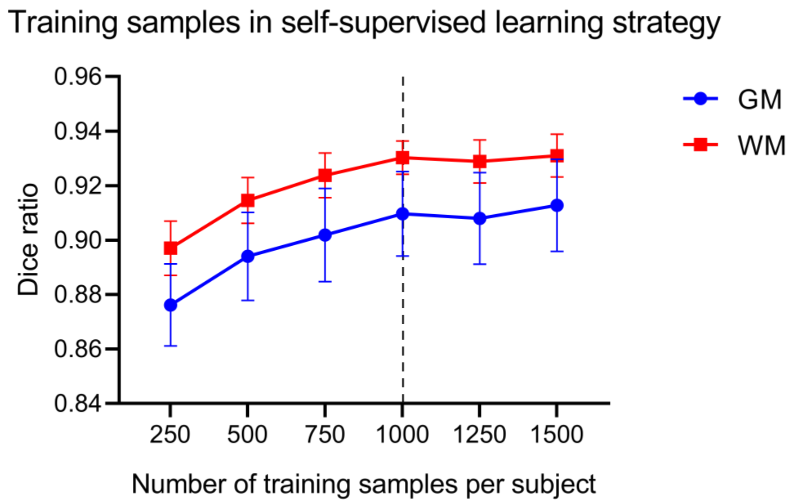

Supplementary Fig. 13. Dice ratio of 10 testing subjects in segmenting GM and WM with different numbers of training samples from each testing subject in the proposed self-supervised training strategy. Error bars represent mean values $\pm$ SD. Source data are provided as a Source Data file.

Supplementary Note 8. Justification of training samples from boundaries

The proposed self-supervised learning strategy generates more reliable labels inside a tissue region compared to those in the boundaries. Supplementary Fig. 14 illustrates the original T1w images from two 6-month-old subjects, the corresponding automated segmentations, confidence maps, and confidence maps after exclusion (by setting the confidence value to 0 if it is less than 0.5). The figure highlights that labels inside a tissue are generally reliable, while labels in the boundaries are less reliable. For example, the confidence value for a boundary voxel A in subject #1 is very low (0.35). However, not all boundary labels are unreliable. For instance, the confidence value for a boundary voxel B in subject #1 is very high (0.98), and it can provide guidance for training the domain-specific segmentation model. Furthermore, we extract training samples from a number of testing subjects. Even if the confidence value at a specific boundary in one testing subject is low (e.g., the voxel A in subject #1), its corresponding confidence values in other testing subjects could be high (e.g., the voxel A' in subject #2 with a confidence value of 0.95). These values provide additional guidance for training the domain-specific segmentation model. Our quantitative analysis on 45 testing subjects from 6- to 18-month-olds demonstrates that the proposed loss significantly outperforms the conventional cross-entropy loss (p-value < 0.05) for most age groups, as shown in Supplementary Table 1 and Supplementary Fig. 11.

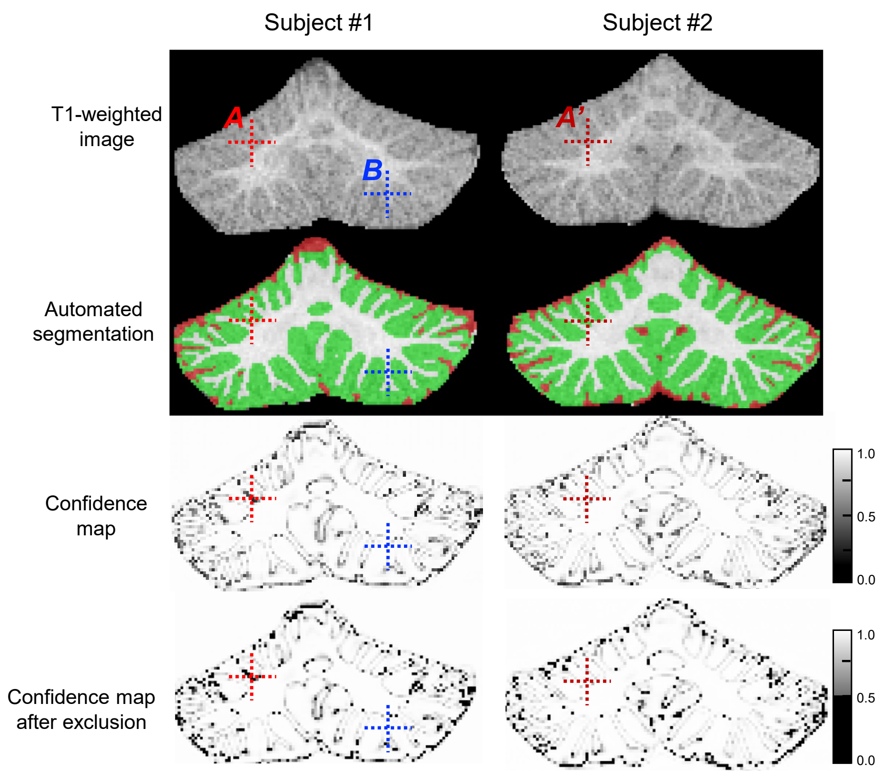

Supplementary Fig. 14. Original T1w images from 6-month-old subjects, corresponding automated segmentations, confidence maps, and confidence maps after exclusion (by setting the confidence value as 0 if it is smaller than 0.5) are shown from the first to the last rows. Confidence values for voxels A, B and A' are 0.35, 0.98, and 0.95 respectively. Automatically-generated labels in the boundaries are still helpful for training, e.g., voxels B and A'.

Supplementary Note 9. Information of the NDAR dataset

For reference, we provide the necessary subject screening information about the NDAR data[3, 4, 5]. Supplementary Table 5 and Supplementary Table 6 list the cerebellar tissue volumes and related clinic measures for 22 autistic subjects and 73 neurotypical subjects from NDAR. The following information is available for each subject:

1. Behavioral assessment: Infants were assessed at 6, 18, and 24 months using the Mullen Scales of Early Learning, the Vineland Adaptive Behavior Scales-II, the Autism Observation Scale for Infants, various questionnaires examining behavior, temperament, family characteristics, and a medical record review. These assessments are available in NIMH Data Archive (https://nda.nih.gov/edit_collection.html?id=19).
2. Site consistency: As reported in[5], the infants were recruited, scanned, and accessed from four clinical data collection sites (University of North Carolina at Chapel Hill, University of Washington, Children's Hospital of Philadelphia, Washington University in St. Louis), a Data Coordinating Center at the Montreal Neurological Institute (McGill University), and two image processing sites (University of Utah and UNC). To ensure the stability of assess scanners and the reliability of brain MRI scans across different sites, time-points, and procedures, a number of quality control procedures were employed, including phantom data evaluation for site stability, and blind review for all scans. Results indicate excellent stability across sites, and two expert raters reviewed all scans to ensure the image quality.
3. As reported in[5], data exclusion criteria during collection include: (a) genetic condition or syndrome associated with ASDs, (b) significant condition affecting development, (c) sensory impairment, (d) birth weight less than $2,000g$ or prematurity less than 36 weeks gestation, (e) possible brain injury during the perinatal period, (f) non-English speaking families, (g) contraindication for MRI, (h) children who do not have a biological relationship with parents or siblings, and (i) first-degree relatives with intellectual disability, psychosis, schizophrenia, or bipolar disorder.

Supplementary Table 2. Available cerebellum tissue segmentation methods for brain MRIs.

Methods	Quantitative evaluation with infant subjects	Number of testing subjects
volBrain[6]	×	-
Infant FreeSurfer[7]	✓	17
SegNet[8]	×	-
FastSurfer[9]	×	-
HighRes3DNet[10]	×	-
PICSL[11]	×	-
FreeSurfer[12]	×	-
SLANT[13]	×	-
AssemblyNet[14]	×	-
ADU-Net[15, 16]	✓	10
Proposed	✓	55

Supplementary Table 3. Statistical difference for subjects used in the cross-sectional analysis and longitudinal analysis.

Cross-sectional Analysis (BCP, 174 neurotypical subjects: 78 male subjects vs. 96 female subjects)												
	Cerebellar GM						Cerebellar WM					
	≤3 months	6 months	9 months	12 months	18 months	24 months	≤3 months	6 months	9 months	12 months	18 months	24 months
Normal[1]?	✓	×	✓	✓	✓	✓	×	×	✓	✓	✓	✓
p-values[2]	0.1048	1.75E-04*	0.0168*	0.0526	8.46E-04*	0.0067*	0.0112*	0.0012*	0.1052	0.0433*	3.05E-04*	0.4081
p-values[3]	0.0825	0.0015*	0.0131*	0.0127*	1.65E-04*	0.0121*	0.0212*	0.0011*	0.1079	0.0307*	7.58E-05*	0.2909
Cohen's d	0.7342	2.0413	1.0541	1.3476	1.3654	0.6854	1.0883	2.1281	0.6549	1.1847	1.4480	0.2462

Longitudinal Analysis (NDAR, 95 male subjects: 22 autistic subjects vs. 73 neurotypical subjects)												
	Cerebellar GM			Growth Rate (Cerebellar GM)			Cerebellar WM			Growth Rate (Cerebellar WM)		
	6 months	12 months	24 months	6→12	12→24	6→24	6 months	12 months	24 months	6→12	12→24	6→24
Normal[1]?	✓	×	×	✓	✓	×	✓	✓	✓	✓	✓	✓
p-values[2]	0.1760	0.0157*	0.0415*	0.1517	0.1055	0.4428	0.1341	0.0130*	0.0264*	0.3543	0.5574	0.6914
p-values[3]	0.2121	0.0445*	0.1080	0.1203	0.1310	0.4787	0.1483	0.0513	0.0805	0.4150	0.4437	0.7506
Cohen's d	0.1874	1.3151	0.2813	0.3473	0.4116	0.1561	0.2443	0.3774	0.3199	0.1985	0.1958	0.0776

	Normalized Cerebellar GM			Normalized Cerebellar WM			Cerebellum			Normalized Cerebellum		
	6 months	12 months	24 months	6 months	12 months	24 months	6 months	12 months	24 months	6 months	12 months	24 months
Normal[1]?	✓	✓	✓	✓	✓	×	✓	✓	✓	✓	✓	✓
p-values[2]	0.2759	0.5634	0.456	0.4298	0.7077	0.9262	0.1647	0.0162*	0.0265*	0.7408	0.8982	0.7011
p-values[3]	0.2700	0.5079	0.3212	0.5460	0.8244	0.9662	0.1682	0.0374*	0.0856	0.7107	0.7890	0.5779
Cohen's d	0.2735	0.1670	0.2477	0.1454	0.0550	0.0104	0.3228	0.4776	0.3907	0.0922	0.0669	0.1399

1 Normal distribution. Jarque-Bera test is used to test the normal distribution.
2 Wilcoxon rank sum test (two-sided).
3 Two-sample t-test (two-sided).
* p-value < 0.05.

Supplementary Note 10. Additional results

As previously mentioned, Supplementary Fig. 15 presents examples of the manual annotations made for BCP subjects. Supplementary Table 2 provides a summary of the available cerebellum tissue segmentation methods for brain MRIs. Moreover, Supplementary Table 3 illustrates the statistical differences observed for the subjects used in the cross-sectional and longitudinal analyses. Finally, Supplementary Table 4 compares the time and memory complexities of different methods during the testing stage.

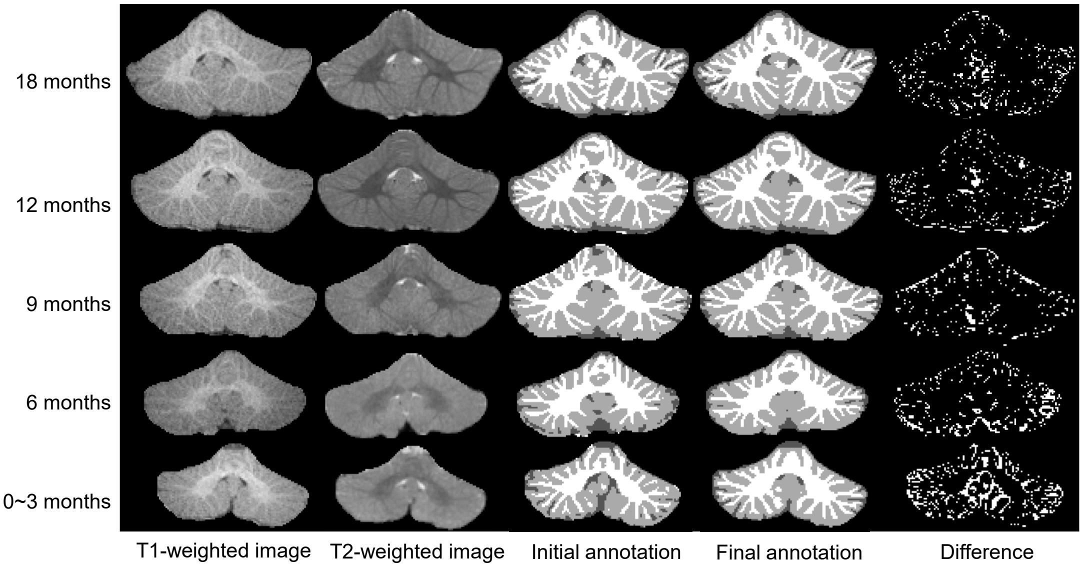

Supplementary Fig. 15. Efforts of manual annotation for BCP subjects from 0∼3 months to 18 months. The first and second columns show the original T1- and T2-weighted images, with the initial and final annotations shown in the third and fourth columns. The last column shows the difference between the initial annotations and the final annotations. Comparing with the initial annotations, for each subject, 21,318±11,411 voxels (10.42%±5.57% of total cerebellum volume) were finally corrected by an infant processing expert and a neuroradiologist.

Supplementary Table 4. **Time and memory complexity during the testing stage for different methods.**

Method	Time	Memory	Note
volBrain[17]	30 min	Not available	Submit to website (https:www.volbrain.upv.es)
Infant FreeSurfer[7]	90 min	6 GB	Processor: 64 x AMD EPYC 7313 16- Core Processor; OS: RedHat 7 x86$_{64}$
Multi-atlas-based method[18]	30 min	10 GB	Processor: 64 x AMD EPYC 7313 16- Core Processor; OS: RedHat 7 x86$_{64}$
ASD-Net[19]	5 min	0.96 GB	Processor: Intel® Core™ i9-8950HK CPU @ 2.90GHz × 12;<br>GPU: NVIDIA GeForce RTX 2080; OS: Ubuntu 18.04.5 LTS
ADU-Net[16]	5 min	1.82 GB	Processor: GNULinux 5.13.044 generic x86_64;<br>GPU: NVIDIA RTX A5000; OS: Ubuntu 20.04.4 LTS
Proposed	5 min	1.82 GB	Processor: GNULinux 5.13.044 generic x86_64;<br>GPU: NVIDIA RTX A5000; OS: Ubuntu 20.04.4 LTS

Supplementary Table 5. **Cerebellar tissue volumes and related clinic measures for 22 autistic subjects from NDAR dataset.**

	Gray Matter (mm^3)			White Matter (mm^3)			Sex	Race	Mullen[20]									ADOS-Module1[21]									
Subject	6 months	12 months	24 months	6 months	12 months	24 months			Date[1]	M1	M2	M3	M4	M5	M6	M7	M8	Date[1]	A1	A2	A3	A4	A5	A6	A7	A8	A9
#1	58322	74173	82814	34980	47641	57633	Male:	White: 90.91%	27	54	66	3	58	3	225	113	3	26	2	1	0	2	8	0	2	2	2
#2	59807	72448	80886	35593	48325	56727	100%	More than one race:	24	20	1	1	20	1	134	69	1	24	2	1	2	1	8	0	1	1	1
#3	57448	70965	77968	36998	47834	54989		9.09%	24	20	1	1	30	1	138	71	2	24	3	1	2	8	8	0	0	1	1
#4	47953	56644	59777	31156	40959	45412			24	63	90	4	46	3	216	108	3	24	0	1	1	0	0	0	0	1	1
#5	52974	73476	82134	34549	49390	56167			24	20	1	1	22	1	106	58	1	24	3	1	0	8	8	1	1	1	2
#6	56007	68835	75997	37108	48851	57255			26	51	54	3	49	3	194	97	3										
#7	45695	54076	60817	26616	36064	43561			24	20	1	1	20	1	80	49	1	24	3	2	8	8	3	8	2	2	2
#8	63036	73057	80231	38232	49750	57879			24	32	4	2	48	3	158	80	2	24	1	0	2	1	1	0	0	1	1
#9	52761	64202	70691	32004	42218	47967			25	20	1	1	36	2	134	69	1	25	3	1	2	0	8	0	0	1	2
#10	59661	68995	74608	36085	47939	55091			26	48	42	3	47	3	189	95	3	26	1	1	0	0	0	0	0	0	0
#11	57567	76262	83403	34782	48762	57849			25	20	1	1	20	1	110	59	1	25	3	3	8	8	8	2	2	1	2
#12	56756	72973	82095	38129	50428	59653			24	47	38	3	48	3	186	93	3	24	1	1	0	0	0	0	2	0	1
#13	63322	79280	86945	41253	54656	63389			24	24	1	1	20	1	92	53	1	24	8	2	2	0	8	0	0	0	3
#14	68346	75537	82117	43951	52961	63038			25	52	58	3	73	5	189	95	3	25	1	1	0	2	0	0	1	1	1
#15	56745	71524	78152	38135	51925	59705			24	50	50	3	48	3	180	90	3										
#16	58478	75760	82677	35302	48994	58602			24	20	1	1	33	2	143	73	2	24	1	1	1	2	1	0	0	1	1
#17	60105	75553	83446	36219	48363	58069			26	20	1	1	40	3	146	74	2	26	2	1	0	0	1	0	0	1	1
#18	49600	55360	60885	33556	41137	48003			24	20	1	1	20	1	84	50	1	24	8	3	1	8	8	9	2	1	3
#19	49416	64723	72083	33226	44664	53231			24	40	16	3	60	3	199	99	3	24	1	1	2	1	8	0	2	1	1
#20	59596	68374	75009	38172	48137	55231			24	56	73	3	38	2	208	104	3										
#21	56885	73987	80762	35915	51762	59709			24	20	1	1	41	3	141	72	2	24	1	1	0	1	8	0	1	1	2
#22	55174	68930	79756	33486	46448	54055			24	20	1	1	28	1	124	65	1	24	8	3	8	8	8	8	2	2	3
Mean	56621	69779	76966	35702	47600	55601	-	-	-	33.5	22.9	1.9	38.4	2.2	153.5	78.9	2.0	-	2.7	1.4	2.1	3.1	4.9	1.5	0.9	1.0	1.6

[1] Age in months at the time of the interview/test/sampling/imaging.
[M1] Receptive Language – T Score.
[M2] Receptive Language – Percentile Rank.
[M3] Receptive Language – Descriptive Category (1=Very Low, 2=Below Average, 3=Average, 4=Above Average, 5=Very High).
[M4] Expressive Language – T Score.
[M5] Expressive Language – Descriptive Category (1=Very Low, 2=Below Average, 3=Average, 4=Above Average, 5=Very High).
[M6] Cognitive T Score Sum.
[M7] Early Learning Composite – Standard Score.
[M8] Early Learning Composite – Descriptive Category (1=Very Low, 2=Below Average, 3=Average, 4=Above Average, 5=Very High).

[A1] Overall Level of Non−echoed Language (ADOS-1 (2001): 0 = Regular use of utterances with two or more words. 1 = Occasional phrases only; mostly single words. 2 = Recognizable single words only; must use at least five different words during session. 3 = At least one word or word approximation, but fewer than five words used during session. 8 = No words or word approximations.).

[A2] Frequency of Vocalization Directed to Others (ADOS-1 (2001) and ADOS-2 (2012): 0 = Directs vocalizations to parentcaregiver or examiner in a variety of pragmatic contexts. Must include chatting or vocalizing to be friendly or to express interest, as well as to make needs known. 1 = Directs vocalizations to parentcaregiver or examiner consistently in one pragmatic context, OR directs a limited number of vocalizations to parentcaregiver or examiner across a variety of pragmatic contexts. 2 = Directs an occasional vocalization to parentcaregiver or examiner inconsistently in a limited number of pragmatic contexts. May include whining or crying due to frustration. 3 = Vocalizations never or almost never appear to be directed to parentcaregiver or examiner, OR rarely or never vocalizes. 9 = Cannot be rated for some reason other than that listed above, such as if an examiner makes an error and does not administer a particular ADOS activity).

[A3] Intonation of Vocalizations or Verbalizations (0 = Normal, appropriately varying intonation, with no peculiar or odd intonation. 1 = Little variation in pitch and tone; rather flat or exaggerated, or occasional peculiar intonation. 2 = Odd intonation or inappropriate pitch and stress, AND/OR markedly flat and toneless mechanical vocalizations, OR an odd cry and few other vocalizations. 8 = NA (insufficient vocalizations for assessment of intonation; includes presence of normal cry and few other vocalizations). 9 = Cannot be rated for some reason other than that listed above, such as if an examiner makes an error and does not administer a particular ADOS activity).

[A4] Immediate Echolalia (ADOS-1 (2001) and ADOS-2 (2012): 0 = Does not repeat others' speech. (Note: Requires at least five words to code 0 rather than 8.) 1 = Occasional echoing. 2 = Echoing words and phrases regularly, but some spontaneous language, which can be stereotyped. 3 = Speech largely consists of immediate echolalia. 8 = No echolalia noted, but language too limited to judge. 9 = Cannot be rated for some reason other than that listed above, such as if an examiner makes an error and does not administer a particular ADOS activity).

[A5] StereotypedIdiosyncratic Use of Words or Phrases (0 = Rarely or never uses stereotyped or idiosyncratic words or phrases. (Note: Requires at least five words to code 0 rather than 8.) 1 = Use of words or phrases tends to be more repetitive than that of most individuals at the same level of expressive language, but not obviously odd, OR occasional stereotyped utterances or use of odd words, OR use of phrases in an unusual way, with other flexible spontaneous language as well. 2 = Often uses stereotyped utterances or odd words or phrases, with some other language. 3 = Frequently uses odd or stereotyped speech, and rarely uses non-stereotyped spontaneous speech. 8 = Language too limited to judge. 9 = Cannot be rated for some reason other than that listed above, such as if an examiner makes an error and does not administer a particular ADOS activity).

[A6] Use of Other's Body to Communicate (0 = No use of another person's body for a specific goal (e.g., to manipulate an object), except in situations where other strategies have not worked (e.g., when others are conversing and the participant cannot get their attention) and in conjunction with coordinated gaze. 1 = Takes another person's hand and leads him/her places without coordinated gaze, but no placement of hand on objects and no use of another person's body as a tool, to point, or for a specific goal. 2 = Movement of another person's hand when it is holding an object OR pushing examiner's hand away from object without eye contact. 3 = Placement of another person's hand or other body part on object (including placing the balloon or food to the examiner's mouth without coordinated gaze), OR use of his/her hand or other body part as a tool or to gesture "for" the participant (such as pointing). 8 = Little or no spontaneous communication.).

[A7] Responsive Social Smile (0 = Smiles immediately in response to one of the first two smiles of the examiner and/or parent/caregiver. This must be a clear change from nonsmiling to a fully responsive smile that is not prompted by a specific request (e.g., "Give me a smile"). 1 = Delayed or partial smile in response to one of the first two smiles of the examiner and/or parent/caregiver, OR smiles fully or partially only after more than two smiles by the parent/caregiver, OR smiles only in response to a specific request. 2 = Smiles fully or partially at the parent/caregiver only after being tickled or touched in some way OR in response to a repeated action with a physical component (even if the child is not actually touched). 3 = Does not smile in response to another person. 9 = Cannot be rated for some reason other than that listed above, such as if an examiner makes an error and does not administer a particular ADOS activity).

[A8] Facial Expressions Directed to Others (ADOS-1 (2001) and ADOS-2 (2012): 0 = Directs a range of appropriate facial expressions to the examiner and/or parent/caregiver in order to communicate affective or cognitive states. 1 = Some direction of facial expressions to the examiner and/or parent/caregiver (e.g., directs only expressions indicating emotional extreme(s) to others, or occasionally directs wider range of expressions). A child who has a limited range of facial expressions, but who directs most of his or her facial expressions to another person, may be rated here. 2 = Does not direct appropriate facial expressions to others. 9 = Cannot be rated for some reason other than that listed above, such as if an examiner makes an error and does not administer a particular ADOS activity.).

[A9] Quality of Social Overtures (0 = Effectively uses nonverbal and verbal means to make clear social overtures to the examiner or the parent/caregiver. The overtures must be appropriate to immediate contexts. 1 = Slightly unusual quality of social overtures. Assign this rating if overtures are restricted to personal demands or related to strong interests, but with some attempt to involve the examiner or the parent/caregiver in those interests. 2 = Overtures often lack integration into context AND/OR social quality. Assign this rating if there are some clearly inappropriate overtures, even if there are other overtures. 3 = No social overtures of any kind. 9 = Cannot be rated for some reason other than that listed above, such as if an examiner makes an error and does not administer a particular ADOS activity).

Supplementary Table 6. **Cerebellar tissue volumes and related clinic measures for 73 neurotypical subjects from NDAR dataset.**

	Gray Matter (mm^3)			White Matter (mm^3)			Sex	Race	Mullen[20]									ADOS-Module1[21]									
Subject	6 months	12 months	24 months	6 months	12 months	24 months			Date[1]	M1	M2	M3	M4	M5	M6	M7	M8	Date[1]	A1	A2	A3	A4	A5	A6	A7	A8	A9
#1	54800	63541	69992	35781	44765	51344	Male:	White: 87.67%	24	59	82	3	73	5	259	129	4	24	0	0	0	1	0	0	0	0	0
#2	56987	67417	74105	36338	46919	53682	100%	More than one race:	28	45	31	3	48	3	197	98	3	28	0	0	0	0	0	0	2	0	0
#3	58126	68878	77791	36553	45638	58317		6.85%	25	59	82	3	57	3	250	125	4										
#4	51528	62514	70976	33064	43871	51288		Black or African American	24	59	82	3	57	3	224	112	3	24	0	0	0	1	1	0	1	1	1
#5	46489	56805	61244	29521	40954	48256		2.74%	24	50	50	3	38	2	178	89	3	24	2	0	1	0	8	0	0	0	1
#6	59573	71249	75335	36778	47099	51304		BA[2] : 1.37%	24	59	82	3	73	5	272	135	5	24	0	0	0	1	0	0	0	0	0
#7	48347	58625	69251	30455	41531	48877		Asian: 1.37%	24	47	38	3	48	3	181	91	3	24	1	0	0	0	0	0	1	0	1
#8	54116	64202	70306	34238	41636	50810			24	59	82	3	51	3	213	107	3										
#9	58861	71332	81463	35450	46121	54332			26	54	66	3	40	3	171	86	3	26	2	1	0	0	8	0	0	0	0
#10	54566	70070	73665	32995	45636	51931			24	47	38	3	51	3	233	117	4	24	1	0	0	1	8	0	0	0	0
#11	53318	67495	73044	34401	44142	53640			25	68	96	4	63	4	239	120	4										
#12	58826	72719	81222	40518	53401	61305			24	56	73	3	46	3	189	95	3	25	0	0	0	1	0	0	0	0	0
#13	63278	79012	86989	40368	53015	63496			24	47	38	3	51	3	207	104	3										
#14	54724	66885	77000	33780	44029	53848			25	46	34	3	30	1	155	78	2	25	2	0	0	1	8	0	0	0	0
#15	55922	63253	71421	34263	43398	51448			26	40	16	3	35	2	176	88	3	26	2	0	0	0	8	0	0	0	0
#16	51553	61892	70411	32487	44108	52447												25	0	0	0	0	0	0	0	0	0
#17	57119	68841	79448	35188	46705	54819			25	54	66	3	45	3	183	92	3	25	0	0	0	0	0	0	0	0	0
#18	52958	66134	77715	32434	43692	51860			24	52	58	3	51	3	189	95	3	24	1	0	0	1	0	0	2	0	0
#19	57892	69979	76307	36190	45981	53299			24	47	38	3	41	3	186	93	3										
#20	54190	64581	71757	35338	48257	55732			26	76	99	5	78	5	314	153	5	26	0	0	1	0	0	0	0	0	0
#21	54576	63155	72229	36113	46916	56292			25	50	50	3	38	2	178	89	3	25	2	1	0	0	8	0	1	0	1
#22	52484	64352	68564	32645	43259	50841			25	54	66	3	40	3	203	101	3	25	1	0	0	0	0	0	0	0	0
#23	55664	67829	71593	34976	47480	54253			24	63	90	4	63	4	269	134	5	24	0	0	0	1	0	0	0	0	0
#24	58633	69018	75738	37232	46173	53261			24	35	7	2	48	3	165	83	2	24	1	0	0	1	0	0	0	0	0
#25	55961	68591	73858	34377	44686	52524			25	37	10	2	45	3	168	84	2	25	1	0	0	0	0	2	0	0	0
#26	54729	70319	78259	32632	46148	53273			24	59	82	3	60	3	222	111	3	24	1	0	0	1	0	0	0	1	0
#27	59678	68534	75437	38035	46153	56114			24	56	73	3	54	3	224	112	3	24	1	0	0	0	1	0	0	1	1
#28	51410	61847	68666	32805	45161	54121			24	66	95	4	54	3	257	128	4	24	1	0	0	0	0	0	0	0	0
#29	56304	73878	82993	36184	51280	60140			25	51	54	3	37	2	193	96	3										
#30	53218	64955	73128	32674	44067	52864												24	0	0	0	1	0	0	0	0	0
#31	56593	72784	80151	34701	48490	56596			25	50	50	3	36	2	209	105	3	25	2	0	0	0	0	0	2	0	0
#32	50795	62123	67825	31996	43249	50716			24	59	82	3	54	3	231	116	4	24	1	0	0	0	0	0	0	0	0
#33	56093	71114	78626	35691	47809	54631			24	59	82	3	57	3	242	121	4	24	0	0	0	0	0	0	0	0	0
#34	52385	64997	69554	30199	41022	47396			24	59	82	3	36	2	193	96	3	24	1	0	0	0	0	0	0	0	0
#35	63117	68039	72984	37865	46007	52441			25	40	16	3	40	3	185	93	3	24	3	1	8	0	0	0	0	0	0
#36	46822	56322	61607	31212	39801	47009			25	47	38	3	44	3	192	96	3										
#37	50359	61050	65899	30638	40918	46630			23	38	12	2	38	2	152	77	2	23	2	0	0	0	8	0	2	1	0

	Gray Matter (mm^3)			White Matter (mm^3)			Sex	Race	Mullen[20]									ADOS-Module1[21]									
Subject	6 months	12 months	24 months	6 months	12 months	24 months			Date[1]	M1	M2	M3	M4	M5	M6	M7	M8	Date[1]	A1	A2	A3	A4	A5	A6	A7	A8	A9
#38	47030	58124	64799	30058	42122	52151			27	34	5	2	35	2	144	73	2	27	2	1	0	0	8	0	0	0	0
#39	59653	74348	84552	36765	48945	60156			24	56	73	3	46	3	196	98	3	24	1	0	0	1	0	0	0	0	0
#40	52187	65941	73450	33199	44846	52928			24	63	90	4	54	3	199	99	3	24	0	0	0	1	0	0	0	0	0
#41	61706	66424	73602	38452	46184	53944			26	54	66	3	74	5	262	131	5	26	0	0	0	0	0	0	0	0	0
#42	54150	66867	74356	33961	46152	54027			24	56	73	3	54	3	220	110	3	24	0	0	0	0	1	0	0	0	0
#43	59237	73739	81107	38434	51574	58560			24	50	50	3	38	2	163	82	2										
#44	50482	64817	71400	33001	44709	54287			25	66	95	4	66	4	225	113	3	24	1	0	0	0	1	0	0	0	0
#45	56204	73474	81478	34097	49299	55541			25	52	58	3	48	3	194	97	3	25	0	0	0	1	0	0	0	1	0
#46	60422	72996	84235	38509	51124	59717			24	52	58	3	54	3	224	112	3	24	1	0	0	1	0	0	0	0	0
#47	59293	73265	80063	38831	53680	63656			24	52	58	3	66	4	233	117	4										
#48	51504	65860	75402	32939	47317	54217			26	54	66	3	40	3	225	113	3	26	0	0	0	1	0	0	0	0	0
#49	50613	61013	69769	31596	42110	48290			25	51	54	3	45	3	182	91	3	25	0	0	0	0	0	0	0	1	0
#50	64172	74458	84552	41883	52750	61861			25	37	10	2	42	3	193	96	3	25	3	2	8	8	8	0	1	1	0
#51	60095	75199	82288	35621	49351	56799			26	57	76	3	58	3	228	114	3										
#52	49897	58315	65652	30144	39650	48064			24	63	90	4	68	4	226	113	3	24	0	0	0	0	0	0	1	0	0
#53	56496	67119	77169	35745	45067	54186			24	47	38	3	46	3	214	107	3	24	1	0	0	0	0	0	0	0	0
#54	60044	64765	70337	37615	46065	50981			24	59	82	3	46	3	211	106	3										
#55	56238	67619	74376	36072	46402	54407			23	63	90	4	51	3	229	115	3										
#56	45915	58922	66052	28770	40641	48629			24	52	58	3	54	3	217	109	3	24	0	0	0	1	0	0	0	0	0
#57	54287	64008	74433	34092	44960	54011			25	56	73	3	46	3	207	104	3										
#58	51404	65604	72544	33381	46821	53773			25	59	82	3	36	2	209	105	3	25	1	0	0	1	0	0	1	0	0
#59	66873	75751	85563	42317	55116	62837			26	48	42	3	52	3	212	106	3	26	1	1	0	0	0	0	1	0	0
#60	56631	65262	73209	34124	43522	49435			26	48	42	3	40	3	167	84	2	26	1	0	0	0	0	0	0	0	0
#61	51539	63416	70227	30956	43152	49773			24	56	73	3	51	3	196	98	3										
#62	62799	73990	81605	38661	50192	58447			25	50	50	3	44	3	207	104	3										
#63	60891	69872	84188	37758	47794	58645			24	71	98	5	70	5	268	133	5	24	1	0	0	0	0	0	0	0	0
#64	63880	78177	84491	38712	49379	57449			24	50	50	3	48	3	177	89	3	24	2	1	0	0	8	0	1	0	1
#65	49606	64256	70038	30173	42883	50907			25	56	73	3	46	3	213	107	3										
#66	61891	74279	82872	39288	49771	56398			26	51	54	3	45	3	204	102	3	26	0	0	0	1	0	0	0	0	0
#67	54777	65498	74411	34836	44970	53386			24	30	2	1	46	3	154	78	2	24	1	0	0	1	0	0	0	0	0
#68	56455	70853	76003	36691	48086	55252			24	68	96	4	60	3	267	133	5	24	1	0	0	0	0	0	1	0	0
#69	57170	65301	74804	36349	46017	54692			25	51	54	3	52	3	213	107	3	24	1	0	0	0	0	0	0	0	0
#70	59399	68516	78297	35892	49326	60352			24	59	82	3	48	3	211	106	3										
#71	60781	75058	82712	36015	47556	55831			24	56	73	3	44	3	213	107	3	24	0	0	0	0	0	0	0	0	0
#72	59329	74674	83117	36519	49903	58990			25	59	82	3	57	3	200	100	3										
#73	50018	57369	66095	29914	39116	45709			24	63	90	4	60	3	249	124	4	24	0	0	0	0	0	1	0	0	1
Mean	55686	67390	75011	34897	46111	54102	-	-	-	53.5	61.2	3.1	50.0	3.0	209.2	104.7	3.2	-	0.8	0.1	0.3	0.5	1.5	0.1	0.3	0.1	0.1

1/M1/M2/M3/M4/M5/M6/M7/M8/A1/A2/A3/A4/A5/A6/A7/A8/A9/a1/a2/a3/a4/a5 Please refer to Table 5 for notations.

Supplementary References

1. Weiyan Yin, Meng-Hsiang Chen, Sheng-Che Hung, Kristine R. Baluyot, Tengfei Li, and Weili Lin. Brain functional development separates into three distinct time periods in the first two years of life. *NeuroImage*, 189:715 – 726, 2019.
2. Y. Sun, K. Gao, Z. Wu, G. Li, X. Zong, Z. Lei, Y. Wei, J. Ma, X. Yang, X. Feng, L. Zhao, T. Le Phan, J. Shin, T. Zhong, Y. Zhang, L. Yu, C. Li, R. Basnet, M. Omair Ahmad, M. N. S. Swamy, W. Ma, Q. Dou, T. D. Bui, C. B. Noguera, B. Landman, I. H. Gotlib, K. L. Humphreys, S. Shultz, L. Li, S. Niu, W. Lin, V. Jewells, D. Shen, G. Li, and L. Wang. Multi-site infant brain segmentation algorithms: The iSeg-2019 challenge. *IEEE Transactions on Medical Imaging*, 40(5):1363–1376, 2021.
3. Nalin Payakachat, J. Mick Tilford, and Wendy J. Ungar. National database for autism research (NDAR): Big data opportunities for health services research and health technology assessment. *PharmacoEconomics*, 34(2):127–138, 2016.
4. Heather Cody Hazlett, Hongbin Gu, Kelly N. Botteron Stephen Dager Robert C. McKinstry, Dennis W.W. Shaw, Martin Styner, Clement Vachet, Guido Gerig, Robert T.Schultz Sarah Paterson, Annette M. Estes, Alan C. Evans, and Joseph Piven. Brain volume findings in 6-month-old infants at high familial risk for autism. *The American journal of psychiatry*, 169(6):601–608, 2012.
5. Robert W. Emerson, Chloe Adams, Tomoyuki Nishino, Heather Cody Hazlett, Jason J. Wolff, Lonnie Zwaigenbaum, John N. Constantino, Mark D. Shen, Meghan R. Swanson, Jed T. Elison, Sridhar Kandala, Annette M. Estes, Kelly N. Botteron, Louis Collins, Stephen R. Dager, Alan C. Evans, Guido Gerig, Hongbin Gu, Robert C. McKinstry, Sarah Paterson, Robert T. Schultz, Martin Styner, null null, Bradley L. Schlaggar, John R. Pruett, and Joseph Piven. Functional neuroimaging of high-risk 6-month-old infants predicts a diagnosis of autism at 24 months of age. *Science Translational Medicine*, 9(393):eaag2882, 2017.
6. Jose E. Romero, Pierrick Coupé, Rémi Giraud, Vinh-Thong Ta, Vladimir Fonov, Min Tae M. Park, M. Mallar Chakravarty, Aristotle N. Voineskos, and Jose V. Manjón. CERES: A new cerebellum lobule segmentation method. *NeuroImage*, 147:916 – 924, 2017.
7. Lilla Zöllei, Juan Eugenio Iglesias, Yangming Ou, P. Ellen Grant, and Bruce Fischl. Infant freesurfer: An automated segmentation and surface extraction pipeline for t1-weighted neuroimaging data of infants 0–2 years. *NeuroImage*, 218:116946, 2020.
8. Vijay Badrinarayanan, Alex Kendall, and Roberto Cipolla. Segnet: A deep convolutional encoder-decoder architecture for image segmentation. *IEEE Transactions on Pattern Analysis and Machine Intelligence*, 39(12):2481–2495, 2017.
9. Leonie Henschel, Sailesh Conjeti, Santiago Estrada, Kersten Diers, Bruce Fischl, and Martin Reuter. Fastsurfer - a fast and accurate deep learning based neuroimaging pipeline. *NeuroImage*, 219:117012, 2020.
10. Wenqi Li, Guotai Wang, Lucas Fidon, Sebastien Ourselin, M. Jorge Cardoso, and Tom Vercauteren. On the compactness, efficiency, and representation of 3d convolutional networks: Brain parcellation as a pretext task. In Marc Niethammer, Martin Styner, Stephen Aylward, Hongtu Zhu, Ipek Oguz, Pew-Thian Yap, and Dinggang Shen, editors, *Information Processing in Medical Imaging*, pages 348–360, Cham, 2017. Springer International Publishing.
11. Hongzhi Wang and Paul Yushkevich. Multi-atlas segmentation with joint label fusion and corrective learning—an open source implementation. *Frontiers in Neuroinformatics*, 7, 2013.
12. Bruce Fischl. Freesurfer. *NeuroImage*, 62(2):774–781, 2012.
13. Yuankai Huo, Zhoubing Xu, Yunxi Xiong, Katherine Aboud, Prasanna Parvathaneni, Shunxing Bao, Camilo Bermudez, Susan M. Resnick, Laurie E. Cutting, and Bennett A. Landman. 3d whole brain segmentation using spatially localized atlas network tiles. *NeuroImage*, 194:105–119, 2019.
14. Pierrick Coupé, Boris Mansencal, Michaël Clément, Rémi Giraud, Baudouin Denis de Senneville, Vinh-Thong Ta, Vincent Lepetit, and José V. Manjon. Assemblynet: A large ensemble of cnns for 3d whole brain MRI segmentation. *NeuroImage*, 219:117026, 2020.
15. Jiawei Chen, Han Zhang, Dong Nie, Li Wang, Gang Li, Weili Lin, and Dinggang Shen. Automatic accurate infant cerebellar tissue segmentation with densely connected convolutional network. *Machine learning in medical imaging. MLMI*, 11046:233–240, 2018.
16. L. Wang, G. Li, F. Shi, X. Cao, C. Lian, D. Nie, M. Liu, H. Zhang, Z. Wu, W. Lin, and D. Shen. Volume-based analysis of 6-month-old infant brain MRI for autism biomarker identification and early diagnosis. In *MICCAI*, volume 11072, pages 411–419, 2018.
17. José V. Manjón and Pierrick Coupé. volBrain: An online MRI brain volumetry system. *Frontiers in Neuroinformatics*, 10:30, 2016.
18. Li Wang, Ken Chung Chen, Feng Shi, Shu Liao, Gang Li, Yaozong Gao, Steve GF Shen, Jin Yan, Philip K. M. Lee, Ben Chow, Nancy X. Liu, James J. Xia, and Dinggang Shen. Automated segmentation of cbct image using

spiral ct atlases and convex optimization. In *MICCAI*, pages 251–258, Berlin, Heidelberg, 2013. Springer Berlin Heidelberg.
19. Dong Nie, Yaozong Gao, Li Wang, and Dinggang Shen. ASDNet: Attention based semi-supervised deep networks for medical image segmentation. In *Medical Image Computing and Computer Assisted Intervention – MICCAI 2018*, pages 370–378, 2018.
20. Laura Shank. *Mullen Scales of Early Learning*, pages 1669–1671. Springer New York, New York, NY, 2011.
21. Catherine Lord, Susan Risi, Linda Lambrecht, Edwin Cook, Bennett Leventhal, Pamela DiLavore, Andrew Pickles, and Michael Rutter. The autism diagnostic observation schedule—generic: A standard measure of social and communication deficits associated with the spectrum of autism. *Journal of Autism and Developmental Disorders*, 30:205–223, 2000.
