# Supplementary material for: Self-supervised learning with application for infant cerebellum segmentation and analysis
Source: Nat Commun. 2023 Aug 5;14:4717. doi: 10.1038/s41467-023-40446-z (PMC10404262; doi:10.1038/s41467-023-40446-z)
Supplement: Supplementary file 3 — Reporting Summary [file 41467_2023_40446_MOESM3_ESM.pdf]

nature portfolio

Corresponding author(s): Li Wang

Last updated by author(s): Jul 5, 2023

Reporting Summary

Nature Portfolio wishes to improve the reproducibility of the work that we publish. This form provides structure for consistency and transparency in reporting. For further information on Nature Portfolio policies, see our Editorial Policies and the Editorial Policy Checklist.

Statistics

For all statistical analyses, confirm that the following items are present in the figure legend, table legend, main text, or Methods section.

n/a	Confirmed	
☐	☒	The exact sample size (n) for each experimental group/condition, given as a discrete number and unit of measurement
☐	☒	A statement on whether measurements were taken from distinct samples or whether the same sample was measured repeatedly
☐	☒	The statistical test(s) used AND whether they are one- or two-sided<br>*Only common tests should be described solely by name; describe more complex techniques in the Methods section.*
☒	☐	A description of all covariates tested
☐	☒	A description of any assumptions or corrections, such as tests of normality and adjustment for multiple comparisons
☐	☒	A full description of the statistical parameters including central tendency (e.g. means) or other basic estimates (e.g. regression coefficient) AND variation (e.g. standard deviation) or associated estimates of uncertainty (e.g. confidence intervals)
☐	☒	For null hypothesis testing, the test statistic (e.g. F, t, r) with confidence intervals, effect sizes, degrees of freedom and P value noted<br>*Give P values as exact values whenever suitable.*
☒	☐	For Bayesian analysis, information on the choice of priors and Markov chain Monte Carlo settings
☒	☐	For hierarchical and complex designs, identification of the appropriate level for tests and full reporting of outcomes
☐	☒	Estimates of effect sizes (e.g. Cohen's d, Pearson's r), indicating how they were calculated

Our web collection on statistics for biologists contains articles on many of the points above.

Software and code

Policy information about availability of computer code

Data collection	No software was used.
Data analysis	The source code for the proposed SSL is available online (https://github.com/DBC-Lab/Self Supervised Learning, https://zenodo.org/record/8050825). In detail, the proposed network is implemented using the Caffe deep learning framework (Caffe 1.0.0-rc3), and the data testing uses the custom Python code (Python 2.7.17). The image preprocessing steps, including skull stripping and cerebellum extraction, was performed by using a public infant cerebrum-dedicated pipeline (iBEAT V2.0 Cloud, http://www.ibeat.cloud). CERES V1.0 pipeline was used to analyze the cerebellum when submitting testing data to volBrain website (https://www.volbrain.net/). We used Infant FreeSurfer pipeline (https://surfer.nmr.mgh.harvard.edu/fswiki/infantFS) updated in Feb 2020 to analyze testing data.

For manuscripts utilizing custom algorithms or software that are central to the research but not yet described in published literature, software must be made available to editors and reviewers. We strongly encourage code deposition in a community repository (e.g. GitHub). See the Nature Portfolio guidelines for submitting code & software for further information.

Data

Policy information about availability of data

All manuscripts must include a data availability statement. This statement should provide the following information, where applicable:

- Accession codes, unique identifiers, or web links for publicly available datasets
- A description of any restrictions on data availability
- For clinical datasets or third party data, please ensure that the statement adheres to our policy

The raw data generated in this study have been deposited in the BCP and NDAR database through standard request procedures (BCP: https://nda.nih.gov/edit collection.html?id=2848/, NDAR: https://nda.nih.gov/edit collection.html?id=19/). The processed Vanderbilt U data are available at https://github.com/YueSun814/Philips data/. The multisite infant cerebrum data used in this study are available at iSeg-2019 challenge (https://iseg2019.web.unc.edu) through standard request procedures. Source data are provided with this paper.

Research involving human participants, their data, or biological material

Policy information about studies with human participants or human data. See also policy information about sex, gender (identity/presentation), and sexual orientation and race, ethnicity and racism.

Reporting on sex and gender	1. For related analyses of cerebellar volumes (i.e., BCP and NDAR datasets), we have indicated the sex, number and age of participants in every experiment. All participating subjects had informed consent provided by their parent or legal guardian. The demographic information of sex and/or gender is listed in public datasets, https://nda.nih.gov/edit collection.html?id=2848/ (BCP), and https://nda.nih.gov/edit collection.html?id=19 (NDAR). We only provide numerical data in aggregate for sex and age information instead of providing such information for each participant.<br>(1) Using BCP dataset (https://nda.nih.gov/edit collection.html?id=2848), we analyse the growth trajectory of infant cerebellums from birth to 2 years old, specifically at 0 months (2M/4F), 3 months (7M/9F), 6 months (7M/9F), 9 months (12M/14F), 12 months (6M/14F), 18 months (17M/20F), and 24 months (27M/26F), where F and M denote female and male, respectively.<br>(2) Using NDAR dataset (https://nda.nih.gov/edit collection.html?id=19), we investigate potential differences in cerebellar growth trajectories between male autistic and neurotypical subjects. Due to the limited number of female autistic subjects, we compare only male autistic subjects with neurotypical subjects using data from NDAR dataset, which includes 95 longitudinal male subjects (22 males with autism and 73 neurotypical males) scanned at 6 months, 12 months, and 24 months of age.<br>2. We do not report sex analysis for testing data acquired with a Philips scanner (i.e., Vanderbilt U data), because we only compare automated cerebellar tissue segmentations between different methods.
Reporting on race, ethnicity, or other socially relevant groupings	We obtained the race classification from a public dataset NDAR. The race information is listed in (https://nda.nih.gov/edit_collection.html?id=19). All participating subjects had informed consent provided by their parent or legal guardian. We only show the race information as numerical data in aggregate in Table SV and Table SVI, to provide the necessary subject screening information about cerebellar analysis related to autism.
Population characteristics	In total 376 male and female subjects were included in this study. The first cohort consists of 276 cross-sectional neurotypical subjects from the BCP, where images were acquired at <=3, 6, 9, 12, 18, and 24 months of age using a Siemens Prisma scanner. The second cohort consists of five 6-month-old MRIs acquired with a Philips scanner in Vanderbilt University. The third cohort consists of 95 longitudinal male subjects (73 neurotypical subjects and 22 autistic subjects) from the NDAR. Each subject was longitudinally scanned at three time-points, i.e., 6, 12, and 24 months of age, where 22 met clinical criteria for autism and 73 did not meet the criteria for autism and were included as neurotypical subjects.
Recruitment	1. According to Howell, B.R. et al. paper [Neuroimage. 185: 891-905, 2019], for the BCP dataset, participants were recruited from existing registries at UNC and UMN based on state-wide birth records as well as from broader community resources (e.g., community centers and targeted day-care centers) to ensure the sample approximates the racial/ethnic and socioeconomic diversity of the US census. To augment recruitment of the youngest cohort of participants, the investigators may recruit participants perinatally by approaching expectant and new mothers at "The Birthplace" at UMN and the UNC Hospitals Newborn Nursery.<br>2. According to information related to the NDAR dataset (https://depts.washington.edu/uwautism/research-projects/infantbrain-imaging-study/), participants were recruited from families with (1) a child between the ages of 4 and 12, (2) an older sibling who is EITHER typically developing OR has an Autism Spectrum Disorder, and (3) who have previously participated in Infant Brain Imaging Study.
Ethics oversight	1. According to Howell, B.R. et al. paper [Neuroimage. 185: 891-905, 2019], for the BCP dataset, all procedures were approved by the University of North Carolina at Chapel Hill and the University of Minnesota Institutional Review Boards.<br>2. According to Hazlett, H. et al. paper [Nature. 542: 348-351, 2017], for the NDAR dataset, data collection sites had study protocols approval from their Institutional Review Boards (IRB), and all enrolled subjects had informed consent provided by parent/guardian.<br>3. For the Vanderbilt U data, all procedures were approved by the Vanderbilt University Institutional Review Board and all participating subjects had informed consent provided by their parent or legal guardian.

Note that full information on the approval of the study protocol must also be provided in the manuscript.

Field-specific reporting

Please select the one below that is the best fit for your research. If you are not sure, read the appropriate sections before making your selection.

- [x] Life sciences
- [ ] Behavioural & social sciences
- [ ] Ecological, evolutionary & environmental sciences

For a reference copy of the document with all sections, see nature.com/documents/nr-reporting-summary-flat.pdf

Life sciences study design

All studies must disclose on these points even when the disclosure is negative.

Sample size

1. For model training, based on our experience and literature, we found 15 subjects would be adequate for training of the proposed work for 24-month-old infants, but the more the better. With 20% size increase, we randomly selected 18 gender-matched subjects from the BCP dataset as the training set.
2. For segmentation comparison experiments (i.e., BCP and Vanderbilt U datasets), due to the difficulty and time-consuming nature of manual annotation editing, a limited number of younger subjects (i.e., ten subjects per time-point in BCP dataset and five 6-month old subjects in Vanderbilt U dataset) were manually annotated for testing data, while the remaining data without manual annotations were visually inspected by two medical students (Yue Sun and Limei Wang).
3. For charting growth trajectories of neurotypical subjects and autistic subjects, no statistical methods were used to predetermine sample size. We have used all available male subjects (i.e., 95) from NDAR, and 258 subjects from BCP. To the best of our knowledge, this might be among the first attempts to compare the GM and WM trajectories of early cerebellum development in terms of gender during the first two postnatal years.

Data exclusions

Data exclusion criteria of the BCP and NDAR during collection are listed in the following:
1. According to Howell, B.R. et al. paper [Neuroimage. 185: 891-905, 2019], for the BCP dataset, subjects are excluded if they were born prior to 37 weeks gestation, had a birth weight lower than 2,000 grams, or if they had any major delivery complications. Major delivery complications may include neonatal hypoxia or neonatal illness requiring a greater than two day NICU stay. They are also excluded if they: (1) are adopted, (2) have a first degree relative with autism, intellectual disability, schizophrenia, or bipolar disorder, (3) have any significant medical and/or genetic conditions affecting growth, development, or cognition, or (4) have any contraindication to MRI. Additional exclusion criteria include major pre- and/or perinatal issues including: (1) maternal pre-eclampsia, placental abruption, maternal HIV status, and maternal alcohol or illicit drug use during pregnancy. Finally, children are excluded from the study if their caregivers are unable to communicate in English at a level to provide informed consent.
2. According to Hazlett, H. et al. paper [Nature. 542: 348-351, 2017], for the NDAR dataset, subjects were enrolled as high familial risk for ASD (HR) if they had an older sibling with a clinical diagnosis of ASD confirmed with the Autism Diagnostic Interview-Revised(ADI-R). Subjects were enrolled in the low familial risk (LR) group if they had an older sibling without evidence of ASD and no family history of a first or second-degree relative with ASD. Exclusion criteria for both groups included the following: (1) diagnosis or physical signs strongly suggestive of a genetic condition or syndrome (for example, fragile X syndrome) reported to be associated with ASDs, (2) a significant medical or neurological condition affecting growth, development or cognition (for example, CNS infection, seizure disorder, congenital heart disease), (3) sensory impairment such as vision or hearing loss, (4) low birth weight (< 2,000 g) or prematurity (< 36 weeks gestation), (5) possible perinatal brain injury from exposure to in utero exogenous compounds reported to likely affect the brain adversely in at least some individuals (for example, alcohol, selected prescription medications), (6) non-English speaking families, (7) contraindication for MRI (for example, metal implants), (8) adopted subjects, and (9) a family history of intellectual disability, psychosis, schizophrenia or bipolar disorder in a first-degree relative. The sample for this analysis included all children with longitudinal imaging data processed until 31 August 2015.
During our data analysis, we have performed tissue segmentation on all the archived subjects from BCP and NDAR. To chart accurate growth trajectory, if any of two raters considered the segmentation quality for one scan as "poor", this scan will be excluded. Finally, we excluded 34 of 493 scans (7%) from BCP and NDAR for charting the growth trajectory.

Replication

The code used for training the deep-learning models are made publicly available for the reproducibility purpose (https://github.com/DBC-Lab/Self_Supervised_Learning, https://zenodo.org/record/8050825). We run the code 3 times with different random initializations for the training samples and there is no statistical
difference.

Randomization

The samples were allocated into experimental groups (training and testing) randomly.

Blinding

For the BCP and Vanderbilt U dataset, the investigators were not blinded to training and testing group allocation since all the subjects are typically developing and the images were de-identified before segmentation and analyses. For the NDAR dataset (autism), the investigators were blinded to diagnosis information before performing the tissue segmentation.

Reporting for specific materials, systems and methods

We require information from authors about some types of materials, experimental systems and methods used in many studies. Here, indicate whether each material, system or method listed is relevant to your study. If you are not sure if a list item applies to your research, read the appropriate section before selecting a response.

Materials & experimental systems

n/a	Involved in the study
☒	☐ Antibodies
☒	☐ Eukaryotic cell lines
☒	☐ Palaeontology and archaeology
☒	☐ Animals and other organisms
☒	☐ Clinical data
☒	☐ Dual use research of concern
☒	☐ Plants

Methods

n/a	Involved in the study
☒	☐ ChIP-seq
☒	☐ Flow cytometry
☐	☒ MRI-based neuroimaging

Magnetic resonance imaging

Experimental design

Design type: N/A.

Design specifications: N/A.

Behavioral performance measures: N/A.

Acquisition

Imaging type(s): Structural MRI.

Field strength: 3 Tesla.

Sequence & imaging parameters: For the BCP dataset, the TR/TE (ms) is 2400/2.2 for T1w images, 3200/564 for T2w images. For the Vanderbilt U dataset, the TR/TE (ms) is 10/4.6 for T1w images, 2500/310 for T2w images. For the NDAR dataset, the TR/TE (ms) is 2400/3.16 for T1w images, 3200/499 for T2w images.

Area of acquisition: A whole brain scan.

Diffusion MRI: ☐ Used ☒ Not used

Preprocessing

Preprocessing software: We performed skull stripping and extraction of the cerebellum by leveraging an infant cerebrum-dedicated processing pipeline (i.e., iBEAT V2.0 Cloud, http://www.ibeat.cloud).

Normalization: For image preprocessing, the resolution of all images was resampled into 0.8 x 0.8 x 0.8 mm^3, and T2w images were linearly aligned with their corresponding T1w images. All images are in their original space.

Normalization template: We randomly selected a subject from the BCP as a template to perform histogram matching for remaining subjects.

Noise and artifact removal: N/A.

Volume censoring: N/A.

Statistical modeling & inference

Model type and settings: No statistical modeling or reference was used in this study.

Effect(s) tested: Cohen's d was used to calculate effect size. No ANOVA or factorial designs were used.

Specify type of analysis: ☐ Whole brain ☐ ROI-based ☒ Both

Anatomical location(s): We performed extraction of the cerebellum and cerebrum by leveraging an infant cerebrum-dedicated processing pipeline (i.e., 1BEAT V2.0 Cloud, http://www.ibeat.cloud). The white matter, gray matter, and cerebrospinal fluid were automatically segmented by our proposed method in this work. We charted the growth traJectories in terms of white matter and gray matter volumes, and their normalized volumes compared with the total cerebellum volume

Statistic type for inference (See Eklund et al. 2016): No inference was used in this study.

Correction: No correction was used in this study.

Models & analysis

n/a	Involved in the study
x	Functional and/or effective connectivity
x	Graph analysis
x	Multivariate modeling or predictive analysis
